# Evaluation of Follicular Synchronization Caused by Estrogen Administration and Its Reproductive Outcome

**DOI:** 10.1371/journal.pone.0127595

**Published:** 2015-05-26

**Authors:** Bi Wu, Yan Shi, Xia Gong, Lin Yu, Qiuju Chen, Jian Wang, Zhaogui Sun

**Affiliations:** 1 Shanghai Medical College, Institute of Reproduction and Development, Fudan University, Shanghai, P. R. China; 2 Key Laboratory of Contraceptive Drugs and Devices of National Population and Family Planning Commission of China, Shanghai Institute of Planned Parenthood Research, Shanghai, P. R. China; 3 Department of Food Science, Shanghai Business School, Shanghai, P. R. China; 4 Department of Assisted Reproduction, Shanghai Ninth People's Hospital, Shanghai Jiaotong University School of Medicine, Shanghai, P. R. China; Institute of Zoology, Chinese Academy of Sciences, CHINA

## Abstract

To evaluate multiple follicular development synchronization after estrogen stimulation in prepubertal mice, follicular responsiveness to gonadotropin superovulation, the prospective reproductive potential and ovarian polycystic ovary syndrome (PCOS)-like symptoms at adulthood, prepubertal mice were intraperitoneally injected with estrogen to establish an animal model with solvent as control. When synchronized tertiary follicles in ovaries, in vitro oocyte maturation and fertilization rates, blastocyst formation rate, developmental potential into offspring by embryo transfer, adult fertility and PCOS-like symptoms, and involved molecular mechanisms were focused, it was found that estrogen stimulation (10μg/gBW) leads to follicular development synchronization at the early tertiary stage in prepubertal mice; reproduction from oocytes to offspring could be realized by means of the artificial reproductive technology though the model mice lost their natural fertility when they were reared to adulthood; and typical symptoms of PCOS, except changes in inflammatory pathways, were not remained up to adulthood. So in conclusion, estrogen can lead to synchronization in follicular development in prepubertal mice, but does not affect reproductive outcome of oocytes, and no typical symptoms of PCOS remained at adulthood despite changes related to inflammation.

## Introduction

Polycystic ovary syndrome (PCOS) also known as hyperandrogenic anovulation (HA) [1] or Stein-Leventhal syndrome [2], is an endocrine disease. About 5% to 10% women of reproductive age (approximately 12 to 45 years old) suffer from PCOS [3], with the most common symptoms of anovulation, excess androgenic hormones, and insulin resistance. Clinically, PCOS patients often exhibit a series of nonreproductive metabolic abnormalities including oligomenorrhea even amenorrhea, obesity, metabolic syndrome, hyperinsulinemia, acne and hissutism [4]. Human ovaries with PCOS enlarge with increased central stroma and numerous peripheral small antral follicles [5]. Although PCOS is thought to be one of the leading causes of female infertility [6, 7] and a severe endocrine problem in women of reproductive age, the mechanism of PCOS has not been clarified. So it need further studies on appropriate animal models [8–11] that mimic PCOS, to discover the related pathogenesis and curative methods [12]. The relationship between ovulation obstacle and endocrine estrogen in PCOS patients has not been identified, at the same time whether the induced follicular synchronization affects future reproductive outcome of oocytes is still inconclusive.

In previous study, we found that estrogen analogues of diethylstilbestrol can promote synchronized follicular development in prepubertal mice [13] to form cysts as in PCOS, though other research suggested that anovulation and cysts caused through estrogen were attributed to the mediation of thymus gland [10]. As we known, estrogen plays an important role in follicular growth, selection and ovulation [13, 14]. Luteinizing hormone (LH) can stimulate theca cells to produce androgen which can turn to estrogen by aromatase catalysis in granulosa cells under the regulation of follicle-stimulating hormone (FSH) [15]. For only small antral follicles begin to have aromatase activity and to produce estrogen by androgen aromatization, estrogen production was thought to start from small antral follicular stage [16]. Accompanied with the increasing of androgen synthesis and aromatase activity, estrogen culminates in tertiary follicles, else with LH and FSH reaching their maxima. The interaction and transformation of FSH, LH, estrogen and androgen are indispensable in a normal ovulatory process. Compared with normal follicles, PCOS had low concentration of estradiol in the follicular fluid and reduced aromatase stock in granulosa cells [17–19]. So the low concentration of estradiol caused by the lack of aromatase is responsible for the possible reasons for the disturbance of follicular development in PCOS. But as pointed out in other research, cultured granulosa cells from PCOS patients produced increased amount of estradiol compared to normal granulosa cells in response to FSH stimulation, suggesting that the intrinsic reaction to FSH was higher in granulosa cells from PCOS than normal control. Considering that the study results above on estrogen did not coordinate, we used estrogen instead of diethylstilbestrol to repeat the relevant experiments on ovarian follicular synchronization. Estrogen is involved in the oocyte development, but its functions involved in the reproductive outcome and PCOS pathogenesis, and possible related molecular pathways need to be determined.

## Materials and Methods

### Ethics Statement

All the experiments were carried out in accordance with the Guidelines for the Care and Use of Laboratory Animals, and were approved by the Ethics Committee of Shanghai Institute of Planned Parenthood Research (SIPPR). The procedures were in accordance with guidelines established by the Ethics Committee of SIPPR. All participants provided written informed consents. Ethical approval was granted by the ethical committee of the SIPPR (2010–11).

Chemicals and reagents used in the present study were purchased from Sigma Chemical Co. unless otherwise specified.

### Animal and the preparation of models

Female C57BL∕6 mice aged 3 weeks were obtained from the SIPPR/BK Laboratory Animal Company (Shanghai, China). All of the mice were caged at controlled temperature of 22–25°C, and under a photoperiod of 14 h light: 10 h dark. All the experimentation was in full compliance with standard laboratory animal care protocols approved by the Institutional Animal Care Committee of Shanghai Institute of Planned Parenthood Research. Female C57BL/6 mice aged 3 weeks were injected intraperitoneally with 10μg/gBW estradiol benzoate (Shanghai General Pharmaceutical Limited company, 2mg/ml) for 5 days to establish model group. In parallel, control group was injected with corn oil, the solvent, of the same volume. Two experiments were planned. Experiment 1: Model mice were sacrificed in the sixth day after estrogen treatment, or at age of 4 weeks as young mouse model of estrogen stimulation on follicular development, with parallel operation for control mice. Experiment 2: young model mice were raised to adult at age of 12 weeks, with the same management for control, to observe the long term effects from the exogenous estrogen treatment.

### Estrous cycle determination in mouse models

Here we use the vaginal smear method as general to determine estrous cycle. The slides with smears were air-dried, and then stained with Giemsa stain for 45 seconds. The slides were rinsed with water, overlaid with a coverslip, and viewed immediately at 200×magnification under bright field illumination. Stages in estrous cycles were determined by occurrences of non-nucleated and cornified epithelial cells, nucleated epithelial cells, and lymphocytes and mucus, as introduced by Felicio, et al [20].

### Detection of estrogen (E2), progesterone (P4), insulin (INS), testosterone (TESTO) and leptin (LEP) in serum by ELISA

To measure E2, P4, INS, TESTO and LEP contents in serum, ELISA was conducted using Mouse E2, P4, INS, TESTO and LEP Elisa kit (X-Y Biotechnology, Shanghai, China) by double antibody sandwich method. Briefly, test standards were diluted to the gradient according to protocol, 50 μl of test standards and 40μl sample dilution were added in duplicate to wells of a micro-titer plate pre-coated with mouse monoclonal antibodies, and then 10μl sample was added into the 40μl sample dilution and incubated for 30 min at 37°C. Next, the liquid was discarded and wells were washed 5 times using the washing buffer. Then 50 μl of corresponding alkaline phosphatase labeled antibody was added to each well and incubated for 30 min at 37°C according to the instruction manuals. After the micro-titer plate was washed, 50 μl of Substrates A and 50 μl of Substrates B were added to each well and incubated for 10 min at 37°C in dark. The optical density was read at 450 nm using a plate reader (BioTek-ELx808, BioTek Instruments, Inc.) within 15 min after the reaction was terminated by adding 50 μl of Stop Solution. The concentrations of E2, P4, INS, TESTO and LEP in serum were calculated according to its respective standard curves.

### Observation of follicular development

Mice were sacrificed by overdose anesthesia. Ovaries were collected, placed in Bouins fixative for 72 hrs, and then stored in 70% ethanol. Fixed ovaries were cleaned of adherent fat, weighed, and embedded in paraffin. Afterwards, they were sectioned at 5 μm, placed on a glass slide, and stained with Harris's hematoxylin and eosin Y (HE staining). The procedure can found in textbooks, briefly as follows. After conventional dewaxing and hydration, sections were stained in hematoxylin dye for 5min; with brief water wash to remove excess dye, then in 1% hydrochloric acid in 75% alcohol for 5sec, and by tap water flushing for 10min and in 2% ammonia for 10min to get bluer; finally by 80% alcohol (5sec) transition, then to 0.5–1% eosin stain for 1min, and through general dehydration of gradient alcohols for slide mounting. Stained sections were examined via light microscopy for the presence of follicular cysts, corpora lutea (CLs), and for follicular dysplasia. Follicular stage recognition and counting were performed according to the published literature [13].

### Ovarian responses to superovulation

Superovulation was applied to both young and adult model mice with an usual procedure, administering 7.5 international units (IU) pregnant mare serum gonadotropin (PMSG) and 46~48 h later 10 IU human chorionic gonadotropin (hCG), both hormones from Ningbo Sansheng Pharmaceutical Co., Ltd. The superovulated mice were killed 13 h after hCG injection, and the oviductal ampullae were opened in M2 medium (Millipore) to release newly ovulated oocyte cumulus complexes (COCs). Then oocytes were denuded of cumulus cells by pipetting in M2 medium containing 0.1% hyaluronidase (Millipore).

### Oocyte developmental potential evaluated by Assisted Reproductive Technologies

Ovaries were place into M2 medium (Millipore), and fixed with a fine forceps under a stereoscopic microscope, punctured follicles with a 1ml disposable syringe needle (FUDA Medical Plastic, Shanghai) to release COCs. The oocyte culture solution (100 ml) was prepared with M199 media (Hyclone) containing 10 mL FBS (Hyclone), 1 ml glutamine (200 mmol/L), 1 ml sodium pyruvate, 1 ml penicillin and streptomycin (penicillin at 10,000 units/ml and streptomycin at 10 mg/ml). For in vitro maturation, we added FSH solution (at final concentration of 0.075 IU/ml, Organon), LH solution (at concentration of 0.075 IU/ml), and hEGF solution (at concentration of 10 ng/mL). The collected oocytes were transferred to maturation medium, placed in the incubator at 37°C in 5% CO_2_. After incubation for 24 hours, mature oocytes in vitro were evaluated routinely, and so were superovulated oocytes.

#### In Vitro Fertilization

Transferred oocytes collected as above into balanced human tubal fluid (HTF, Millipore). Masses of dense sperm were collected from the cauda epididymis of fertile male mice and were placed at the 4 well plate (Thermo Scientific™ Nalgene™ labware, USA) containing HTF medium covered with mineral oil and balanced in 37°C, 5% CO_2_ for 3 hours before used. Incubated the plate in 37°C, 5% CO_2_ for 30~60 min to make sperm capacitation. Then 100μl capacitated sperm were added to the oocytes drop to fertilize. Oocytes were observed under a stereomicroscope for fertilization at 6 h after insemination. Oocytes showing two pronuclei and two polar bodies were considered fertilized.

#### Embryo Culture

Fertilized oocytes were cultured for 4 days in KSOM (Millipore) (20~30 oocytes per 100μl drop) at 37°C under humidified atmosphere with 5% CO_2_ in air.

#### Embryo Transfer

Female ICR mice of 8~10 weeks old were paired with vasectomized males to allow mating. The females were checked for vaginal plugs the next morning, and those showing a vaginal plug were used for pseudopregnant recipients. Embryos at 2-cell stage were transferred to the oviduct on Day 0.5 post coitus. Blastocyst transfer to the uterus was done on Day 2.5 post coitus. After the embryo transfer, the recipients were caged singly until parturition.

### Immunohistochemistry

Ovaries were directly fixed in 4% paraformaldehyde for 72 h, dehydrated in gradient alcohol, and followed by embedding in paraffin. Sections (5μm) of ovarian tissues were deparaffined and rehydrated. The immunohistochemical procedure was outlined as follow. For antigen retrieval, the sections were boiled in a microwave oven (800 W) in 10 mmol/L citrate, pH 6.0 for 2 times of 5 min each, and then washed in PBS for 3 times of 5 min. Incubation with 3% H_2_O_2_ in PBS to abolish the endogenous horse radish peroxidase (HRP) activity. For immunohistochemical detection, after non-specific binding blockade for 1 h with 10% normal goat serum in PBS, sections were incubated with rabbit anti-FSHR (1:50) (BOSTER), rabbit anti-LHR (1:50 dilution) (BOSTER), or rabbit anti-LepR (1:100 dilution) (abcam) in 10% normal bovine serum diluted with PBS overnight at 4°C. After washing in PBS three times of 5 min each, sections were incubated with biotinylated goat anti-rabbit IgG (1:200 dilution) (AOGMA) 37°C for 1h. After washing in PBS three times, each for 5 min, sections were incubated with Peroxidase-conjugated Streptavidin (1:200 dilution) (Protein Tech) 37°C for 1h, followed by Vector NovaRED Substrate Kit (Vector Laboratories) according to the manufacture’s protocol. Omitting the primary antibodies was used as control for immunohistochemical method. Sections were counterstained with haematoxylin and mounted regularly. The degree of staining was assessed through examination on two slides by two investigators independently.

### Western Blotting

Frozen ovarian tissues were thawed in ice-cold protein extraction buffer (EB: 50 mmol/L HEPES (pH 7.5), 100mmol/L NaCl, 10 mmol/L MgCl2, 25 mmol/L β-glycerophosphate (Merck), 1mmol/L Na3VO4, 50 mmol/L NaF, 1mmol/L EDTA, 0.5 mmol/L EGTA; 10 mg/mL each of soybean trypsin inhibitor (Sigma), leupeptin and aprotinin (Amresco); and 1 mmol/L PMSF (Sigma)), then homogenized by the glass dounce homogenizer. Solutions were subsequently centrifuged at 14000 g for 10 min at 4°C, and the supernatant was collected as the total protein extract. Protein concentration of each total protein extract sample was determined by Bradford assay. Following a described protocol, 30 μg total protein extracts of each sample and LMW SDS-PAGE Markers (Tanon) were subjected to reducing 10% sodium dodecyl sulfate-polyacrylamide elelectrophoresis (SDS-PAGE), and proteins were transferred onto a nitrocellulose membrane (Immobilion TM-NC, Millipore Corporation, Billerica). The membrane was incubated with blocking solution (6% no-fat milk powder in TBST with 0.05% Tween-20 (Sigma)) for 1 h, and subsequently, incubated respectively with the rabbit anti-FSH Receptor sera (1:400, BOSTER), or the rabbit anti-LH Receptor sera (1:250, BOSTER), or the rabbit anti-Leptin Receptor sera (1:2000 diluted in blocking solution, Abcam) overnight at 4°C. Then the membrane was washed and incubated with goat anti rabbit IgG-HRP (1:2 000 diluted in blocking solution, Invitrogen) for 1 h at room temperature. After further washing, the immunoreactive complexes on the membrane were visualized by staining with SuperSignal West Dura Extended Duration Substrate (Thermo) according to its user’s manuals. Rabbit anti-GAPDH sera (1:2000, Proteintech) was applied as a sample loading control.

### Different gene expressions in mRNA profiles

Total RNA was extracted from ovaries using TRIzol (Invitrogen). Total RNA quality was confirmed through agarose gel electrophoresis, and 2 μg of total RNA was used for purification of mRNA, which was used to establish cDNA library. The cDNA library preparation procedure mainly referenced to the protocol of Genergy Bio, Shanghai China. Sequencing of cDNA samples was conduct on Illumina Hiseq2500, 50bp sequence reads were obtained. The reads were trimmed and then mapped to the whole genome. All differentially expressed genes between two samples were analyzed by Cuffdiff Program based on FPKM value. GO and KEGG pathway analysis were conducted on the differentially expressed genes to illustrate their functions. On the basis of sequencing results, some noticeable differentially expressed genes were validated by quantitative RT-PCR methods.

### Statistical analysis

SPSS 18 was used for the data analysis. Independent-samples t-test was used to estimate the statistical difference between groups. A value of P < 0.05 was considered significant.

## Results

### Possible ovarian follicular abnormalities and oocyte development potential evaluation in the mouse model derived from exogenous estrogen stimulation

**Fig 1:**
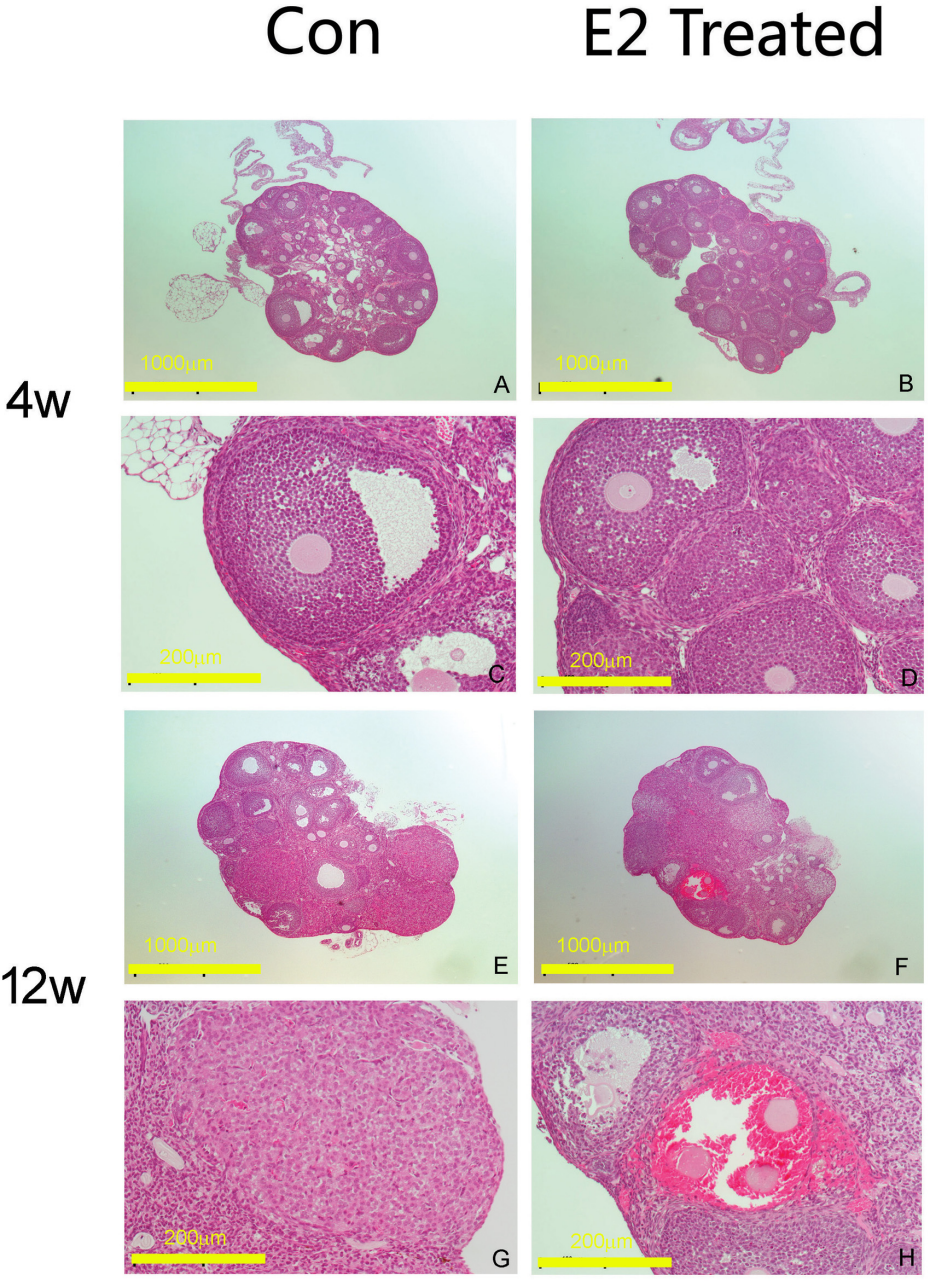
HE staining of mouse ovarian sections. Panel A and C, Ovarian sections of control mice at the age of 4 weeks; Panel B and D, Ovarian sections of estrogen treated mice at the age of 4 weeks, illustrating a synchronization phenomenon of follicular development. Panel E and G, Ovarian sections of control mice at the age of 12 weeks; Panel F and H, Ovarian sections of estrogen treated mice reared to adult of 12 weeks old, illustrating fewer corpora lutea and occasional follicles with blood cell invasion. The scale bar is indicated at individual pictures, in response to two different amplifications. The amplification folds of panel A, B, E and F is 40×. The amplification folds of panel C, D, G and H is 200×.

Mice 21 days after birth were intraperitoneally administrated with estrogen for 5 days consecutively, and samples were collected on the sixth day. The observation on ovarian sections revealed that the numbers of tertiary follicular increased, showing a synchronization phenomenon of follicular development, compared with control (Fig 1, row 1 and 2 indicated as 4W).When model mice induced by estrogen stimulation above were reared to adulthood, no obvious difference was found in the ovarian follicular development and spontaneous ovulation (Fig 1, row 3 and 4 indicated as 12W). Only in a few of individual ovaries, corpora lutea became fewer, and blood cell invasion into follicles were observed occasionally (Fig 1, panel F and H). After observation of consecutive sections, the number of tertiary follicles in each ovary was evaluated at 184.0±6.2 in estrogen treated young mice, significantly higher than that in control, 82.0±20.4. After manual operation, significantly more oocyte cumulus complexes were isolated (Table 1), designated as retrieved oocytes, 122.0±25.0 vs 24.0±6.0.

**Table 1:** Estrogen treatment on prepubertal mice increases the number of tertiary follicles and promotes oocyte development in vitro.

Mouse models	Follicles per ovary(n = 4)	Retrieved oocytes	Maturated oocytes	Maturation rate(%)	Fertilization rate(%)	ART outcome
E2 treated (n = 3)	184.0±6.2*	122.0±25.0*	61.0±41.0*	47.6±23.6%	17.1±19.8%*	0/30
Con (n = 4)	82.0±20.4	24.0±6.0	10.0±4.0	38.4±4.8%	43.0±30.1%	3/30

* indicated the data is different from Con at a statistically significant level <0.05 using independent sample t test. ART, abbreviated from artificial reproductive technology.

Under in vitro conditions, a larger number of oocytes underwent induced maturation (61.0±41.0 vs 10.0±4.0), but no significant difference in its percentage was distinguished. Noticeably, fertilization rate was significantly decreased (17.1±19.8% vs 43.0±30.1%) for oocytes from exogenous estrogen stimulated young mice. Further by transferring embryos into pseudopregnant mouse uteri, 10 embryos were transferred in a single uterine horn of each mouse, only 2 mice in control group of 3 recipients were resulted in pregnancy, with 2 and 1 normal offspring born respectively; and embryo transfer in the estrogen treatment group did not produce pregnant mice (Table 1).

When estrogen treated mice were raised to adult, and mated with intact males, only two occurred with pregnancy among 18 mice which had been observed vaginal semen plugs. One of the pregnant mice bore a normal offspring and the other happened miscarriage; but in control group, 17 mice obtained normal pregnancy among 18 successfully mated females. So in the model group, adults were of normal characteristics in ovulation and estrous cycles, but normal pregnancy could not be obtained possibly due to uterine edema caused by exogenous estrogen stimulation (photos in Supplementary materials).

### Superovulation of estrogen treated mice and their oocyte development potential evaluation

When estrogen treated mice were given the routine superovulation treatment from 4 weeks of age, there was no significant differences in numbers of oocytes obtained from the fallopian tubes, and in developmental percentages at different stages up to blastocysts, as compared with control (Table 2). But when the estrogen treated mice were reared to adult (12 weeks old), the artificial superovulation on them produced similar consequences, and as well no significant difference was found between the model and control groups. So it was found that the ovulation number and ongoing developmental potential of oocytes did not change significantly in response to estrogen treatments in both young and adult mice (Table 2 and S1 Table). When oocytes obtained by the superovulation underwent in vitro fertilization and cultured to two cell stage embryos or blastocysts, they were transplanted into unilateral fallopian tubes or uterine horns of pseudopregnant mice, the result showed that 3 out of 52 two-cell embryos, and 16 out of 31 blastulas developed into normal offspring as for oocytes from young mice; in the case of adults, only 8 out of 76 two-cell stage embryos produced progeny (Table 2 and S1 Table).

**Table 2:** Effects of Estrogen stimulation on superovulation number and oocyte developmental potential in vitro.

Mouse models		Superovulated oocytes per mouse	Percentage of 2-cell embryos	Percentage of 4–8 cell embryos	Percentage of blastulae	ART outcome
Young	E2*treated	31.3	90.02±2.1	85.70±3.0	82.18±5.1	19/83
	control	28.6	89.50±1.9	84.72±1.1	81.14±3.7	
Adult	E2 treated	25.4	89.64±1.3	83.34±1.7	76.14±5.6	8/76
	control	23.9	90.92±2.7	85.02±2.8	81.20±4.3	

* All data with E2 treatment were evaluated at statistically non-significant differences from control using independent sample t test (p>0.05). ART, abbreviated from artificial reproductive technology.

### The estrous cycle and focused hormone serum levels of estrogen stimulated mice

Vaginal smears to last for 13 consecutive days were taken on 12 weeks old mice to check the estrous cycle by observing morphological characteristics of different cell types under microscope routinely, and the result showed estrogen stimulated adult mice have normal estrous cycles, similarly as control. Only insulin level was reduced significantly while serum levels of leptin, testosterone, progesterone and estrogen were detected with non-significant changes (Table 3).

**Table 3:** Blood hormone levels in estrogen stimulated 12 weeks old mice.

	E2 treated	Con	p value
INS(mIU/L)	5.59±0.12	6.10±0.20	<0.05
LEP(pg/ml)	429.60±8.16	439.20±15.03	>0.05
TESTO(nmol/L)	7.28±0.17	6.95±0.48	>0.05
P4(pmol/L)	1492.72±44.58	1406±103.33	>0.05
E2(pmol/L)	37.19±1.53	34.17±1.23	>0.05

All data with E2 treatment were evaluated at statistically significant differences from control using independent sample t test.

### Immunohistochemical detection of FSHR, LHR and LepR in 4 week old and 12 week old mice ovaries

**Fig 2:**
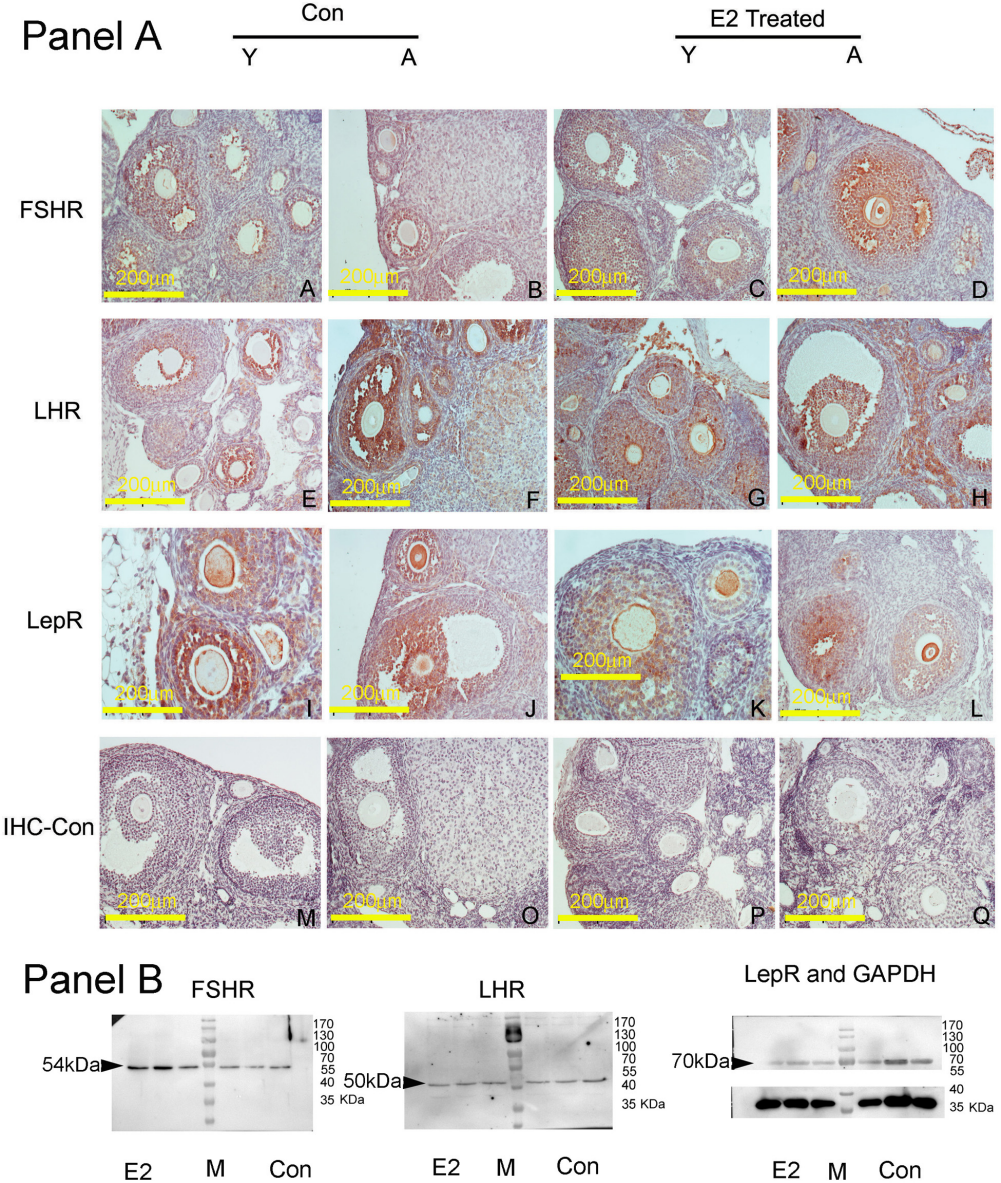
Immunohistochemical and Western Blotting detection of FSHR, LHR and LepR protein in mouse ovaries. In Panel A, Columns of pictures show results on mouse ovaries of different groups, of which Con-Y indicates control mice 4 weeks old; Con-A, control mice of 12 weeks old; and E2 Treated-Y indicates estrogen treated mice of 4 weeks old; and E2 Treated-A, estrogen treated mice reared to 12 weeks old. As marked at left, different rows offer results on corresponding receptors, and IHC-Con indicates immunohistochemical negative controls. The scale bars in the individual pictures equal to 200 μm, and all the amplification folds is 200×. In Panel B, different receptor protein levels were detected by the Western Blotting method in ovarian tissues. Pictures from left to right showed FSHR, LHR and LepR protein bands in turn, and the molecular weights of target bands are about 54, 50 and 70kDa respectively. Under the LepR picture was the sample loading control, with GAPDH protein as a reference. E2 and Con indicated ovarian tissues of adult mice in estrogen treatment model and control groups respectively, with repeats of three mice; M expressed protein molecular weight standards.

As shown in Panel A of Fig 2, the expression of FSHR, LHR and LepR in ovaries of model and control mice at the ages of 4 and 12 weeks old were checked with immunohistochemical methods. It was observed that FSHR, LHR, and LepR proteins present in ovarian tissue specifically. As shown in Fig 2, since the beginning of secondary follicular stage, FSHR appeared in granulosa cells, especially those close to oocytes; at the same time, occurred in the oocyte membrane of adult mice more abundantly in estrogen treatment group than in control (Fig 2, Panel A, B, C and D). LHR expressed strongly in granulosa cells from secondary follicular stage, gently in corpus lutea, and rarely in oocytes (Fig 2, Panel E, F, G and H). Noticeable, in follicles near the medulla of ovaries, positive signals of the two kinds of receptors above had not decreased owing to estrogen treatment. As for LepR, its protein occurred similarly in granulosa cells as LHR, but differently, abundantly in oocytes, especially in their membrane. (Fig 2. Panel I, J, K and L). Relatively, LepR was more abundant at age of 4 weeks than 12 weeks. On IHC control, omitting primary antibodies, no positive signal was observed (Fig 2. Panel M, O, P and Q).

In Panel B of Fig 2, the Western Blotting results showed that estrogen treatment resulted in a significant increase in concentration of FSHR protein in ovarian tissues, while no significant changes were observed in levels of the two other receptor proteins LHR and LepR.

### Differentially expressed genes in ovaries of estrogen treated mice at the ages of 4 weeks and 12 weeks

**Fig 3:**
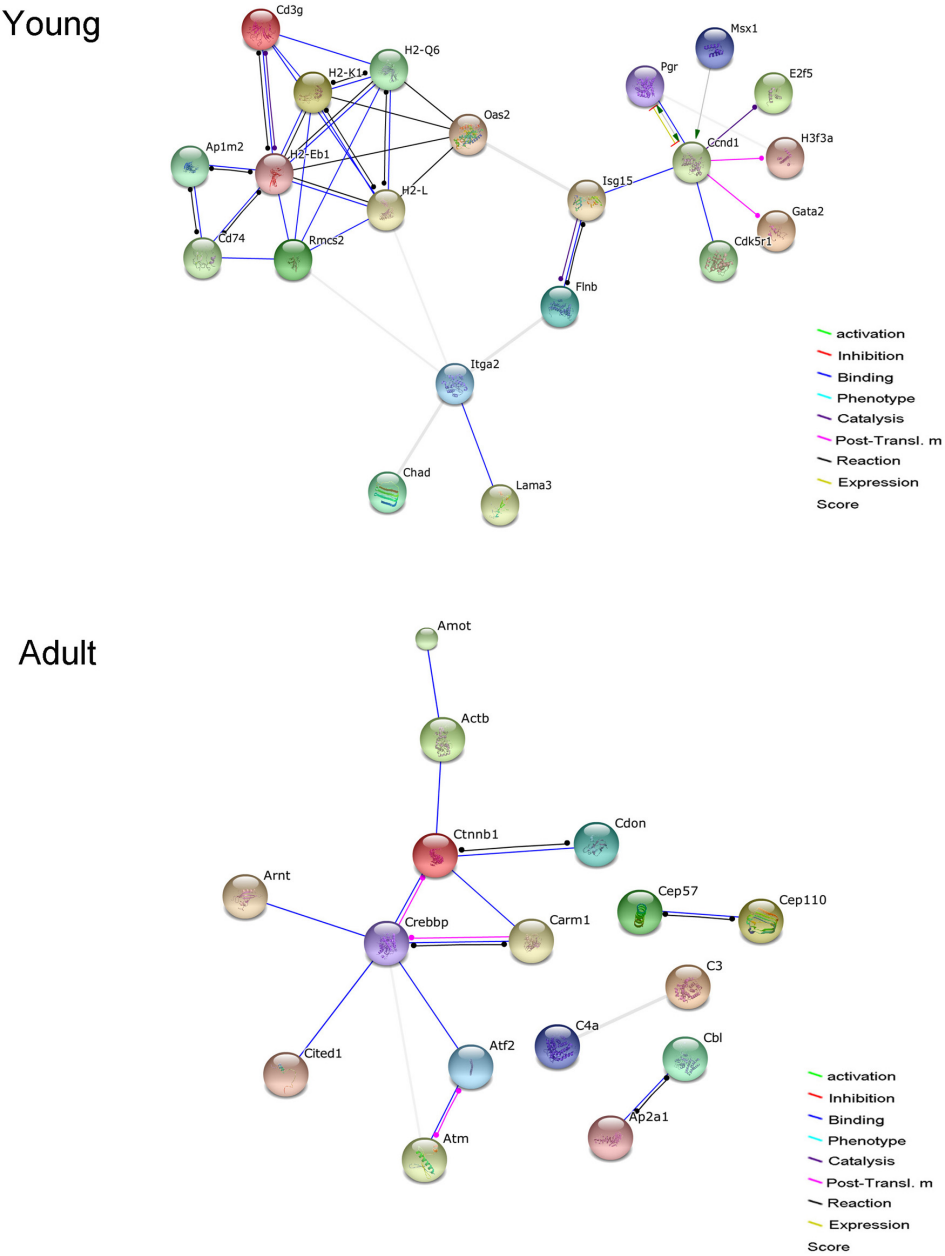
Associations of gene expression changes resulted by estrogen treatment in mice. Using program String 9.1, associations were drawn among the corresponding proteins with different gene expressions between the model of estrogen treatment and control. Association modes were annotated at the right bottom, required confidence selects 7.

Through high-through analysis, we found a large number of candidate genes involved in the estrogen stimulation. Estrogen treatment leads to significant differences in the expression of 544 genes in prepuberty, and when reared to adulthood, there were still 365 genes with differential expression. We analyzed the biological processes and pathways involved, and significant pathways in prepuberty includes metabolism of xenobiotics by cytochrome P450, drug metabolism-cytochrome P450, metabolic pathways, staphylococcus aureus infection, leishmaniasis, complement and coagulation cascades, steroid hormone biosynthesis, phagosome, drug metabolism-other enzymes, antigen processing and presentation and PPAR signaling pathways etc.; and in adulthood, bacterial invasion of epithelial cells, TGF-beta signaling pathway, ubiquitin mediated proteolysis etc. With the aid of the String 9.1 program, progesterone receptor Pgr was found to be associated with proliferation and immunity related genes in prepuberty; and associations of genes with Crebbp as the center, and those of C3 related genes were deduced in the adulthood (Fig 3 and S2 Table). Some key genes were validated by quantitative real-time RT-PCR, the results show the changing trend of selected genes, but with less degrees of gene expression changes (S3 and S4 Tables). For reference, we attached all the gene lists with significant expression changes (S5 Table).

## Discussion

According to a study on Zebu cows (Bos indicus), the quality of cumulus-oocyte complexes (COCs) was not affected by the presence of a single dominant follicle, but co-dominant follicles resulted in recovery of a lower proportion of viable embryos and a higher proportion of degenerate COCs during ovum pick-up, suggesting that synchronized development of more than one dominant follicle may affect the future dominant follicles [21]. As reviewed early [22], follicular growth in women starts when the secretion of FSH and LH rises during luteal regression at the end of each menstrual cycle, and the developing follicles are the major source of estrogen. The dominant follicle secretes estradiol stimulating its own growth while at the same time (by its effects on the hypothalamic-hypophysial axis) inhibiting further growth of other developing follicles, which consequently become atretic. Development of dominant follicles resulted in more estrogen, and caused FSH dependent growth inhibition of non-dominant follicles of smaller diameter through feedback inhibition to pituitary FSH secretion. That shows that the rise of estrogen serum levels maintains the dominant follicle and to inhibit growth of non-dominant follicles. Our results showed that exogenous estrogen applied through intraperitoneal injection lead to synchronized growth of over-numbered ovarian follicles in prepubertal mice, resulting in occurrence of a large number of early tertiary follicles.

However, the reasons why estrogen induced synchronized growth of more follicles could be attributed to intra-ovarian signaling pathways. Usually, feedback inhibition of estrogen to hypothalamic FSH secretion leads to stagnation of follicular development, instead of promotion, so synchronized follicular growth caused by administrated estrogen is not possible due to the feedback action of estrogen on hypothalamus, but direct effects on ovaries. Serum estrogen levels did not increase significantly in the synthetic estrogen diethylstilbestrol (DES) treated mouse models by intraperitoneal administration, suggesting that estrogen signaling feedback to the hypothalamus is not critical, at least not an exclusive pathway (data not included), and possibly estrogen acted directly on ovaries to cause developmental synchronization of ovarian follicles. Estrogen can increase sensitivity of FSH through up-regulation of FSH receptors in follicles so that follicular development initiated in response to low concentration of FSH. Immunohistochemical results on changes in their distribution and abundance of FSHR, LHR and LepR in ovarian follicles after estrogen treatment showed that FSHR increased in the oocytes and the involved tertiary follicles, supporting that estrogen directly promoted follicular growth independent of hypothalamus and pituitary. Another study on zebrafish follicles in vitro demonstrated that 17-beta-estradiol (E2), but not testosterone, was a potent endocrine hormone that differentially regulated the expression of FSHR and LHR. In the presence of oocytes, estrogens enhanced FSH-induced increases in FSH receptor mRNA expression through GRK-6 (G protein-coupled receptor kinase-6) in rat ovarian granulosa cells [23]. E2 likely acted at the transcription level via its nuclear estrogen receptors (ERα and ERβ), because ICI 182,780 (an estrogen receptor inhibitor) could abolish its effects, but other evidences suggested that these receptors might be localized on the plasma membrane [24]. When transiently transfected cDNAs for ERα and ERβ into Chinese hamster ovary (CHO) cells respectively, both membrane and nuclear ERs can be derived from a single transcript and have near-identical affinities for 17-beta-E2, and membrane ERs derived from both ERα and ERβ can activate G proteins, ERK, and cell proliferation [25]. Moreover, studying on cultured cumulus-oocyte complexes (COCs) isolated from mice [26], it was found that estradiol promotes and maintains expression of natriuretic peptide receptor 2 (NPR2) in cumulus cells and participates in natriuretic peptide type C (NPPC)-mediated maintenance of oocyte meiotic arrest in vitro. In short, estrogen can act directly on the ovary via different pathways to increase FSH sensitivity by up-regulating FSHR expression in follicular granulosa cells, or via its membrane receptors to promote rapid cell proliferation or oocyte development. In this study estrogen administrated by intraperitoneal injection resulted in synchronized development of prepubertal mouse ovarian follicles by increasing FSH sensitivity to promote synchronous growth of over-numbered follicles to arrest in the early tertiary stage via its membrane receptor functions.

Moreover in this study, although we found that exogenous estrogen stimulation on prepubertal mice increased the number of tertiary ovarian follicles, routine superovulation treatments were not able to change significantly the oocyte harvest from oviducts relative to control, indicating that the tertiary follicles induced by exogenous estrogen did not respond to superovulation stimulation as spontaneous tertiary follicles. In our results, FSHR, LHR, and LepR all maintained higher levels in the tertiary follicles, so it was thought that there was no response to superovulation stimulation of those follicles driven by exogenous estrogen was not attributed to these receptors. As for oocytes, no significant damage had been observed to those COCs obtained by puncture operations, and thereafter at the stages of in vitro maturation and fertilization, early embryo culture to blastocyst; but we did not determine whether any potential impact had been added into the oocytes, on embryo implantation and the later gestation, even though no live offspring was produced in the estrogen treated group, because the decreased embryo implantation rates could result from the applied artificial assisted reproductive technology itself. In addition, obtaining oocytes from fallopian tubes after the artificial superovulation in young mice, undergoing in vitro fertilization and embryo culture, and transferring of two-cell stage embryos into fallopian tubes or blastocysts into a single uterine horn, normal offspring were generated, indicating that exogenous estrogen stimulation did not damage significantly on the superovulated oocytes, possibly not as in the situation of oocyte collection by operation, in which a larger number of oocytes were harvested. So we think that exogenous estrogen caused follicular developmental initiation of over-numbered oocytes, but those ovarian follicles did not achieve to the growth stages which can respond to gonadotropin to undergo ovulation.

When raised to adult, estrogen treated mice showed no obvious symptoms of PCOS, without polycystic ovaries but with spontaneous ovulation. As an explanation, when estrogen was injected prior to 10-days of age, it could act on the thymus and caused anovulation and follicular cysts [27], but beyond the sensitive period of thymus, such as after 21-days of age, it will no longer produce such effects that ovulation was inhibited and follicular cysts had developed. Mating estrogen treated mice with intact males, rare spontaneous pregnancy was observed in this study, significantly different from control. Its main causes could not be attributed to oocytes themselves, but to the uterine edema resulted from estrogen treatment, supporting by the morphological comparison and the fact that normal offspring could be obtained from the superovulated oocytes with assisted reproductive technology. So in summary, effects of estrogen on ovaries depends on the thymic sensitive period causing typical symptoms of PCOS in adulthood, but the subsequent effects on uteri, not on oocytes, caused adult infertility. Because estrogen doses we used were much higher than the physiological level, so we believe that in clinic uterine edema can avoided by controlling estrogen doses. However, in our mouse model, mechanisms involved in the uterine edema worth further study.

Intraperitoneal estrogen treatment, at the dosage of 10 μg/g BW per day and for five consecutive days from the age of three weeks, resulted in accumulation of early tertiary follicles in the prepubertal ovaries. As mentioned above mainly owing to its direct effects on ovaries. Then, what were the molecular signaling pathways involved? The gene transcription profiles in this study showed that a series of genes related to cell proliferation were up-regulated, and other genes related to apoptosis were down-regulated by exogenous estrogen in prepubertal mouse ovaries. At the same time, pathway analysis showed significant differences between model and control groups, which included steroid hormone biosynthesis, cell proliferation, inflammatory reaction pathways, and etc. For a large number of related genes could not act alone, we used the database information to find the associated genes under the published data to explain the involved molecular mechanisms. The expression differences in genes related to steroid hormone biosynthesis, cell proliferation and apoptosis, which were scanned out at prepuberty did not continue into adulthood, but some changes related to inflammatory reactions remained at adulthood. With the help of internet software string 9.1, associations of involved proteins were summarized according to previous studies. A possible signaling pathway related to cell proliferation mediated by progesterone receptor (PR) can be extracted, which may underlie growth initiation of follicular granulosa cells. In clinical assisted reproductive procedure, human menopausal gonadotropin (hMG) was applied during the luteal phase to stimulate follicular growth possibly by up-regulating luteal progesterone effects [28], though the accumulation of PR in dominant follicles needed to be determined. At the same time, another possible signal pathways related to inflammatory reactions continued to adulthood in ovaries as in uterine edema though prepubertal synchronized follicles did not extend into adulthood to form significant PCOS symptoms. As included in the protein relationship, E2f5, E2F transcription factor 5 Gene, as a transcriptional activator, binds to E2F sites, and these sites are present in the promoter of many genes whose products are involved in cell proliferation. Isg15, SG15 ubiquitin-like modifier Gene, as a ubiquitin-like protein, it is conjugated to intracellular target proteins after IFN-alpha or IFN-beta stimulation, which include STAT1, SERPINA3G/SPI2A, JAK1, MAPK3/ERK1, PLCG1, EIF2AK2/PKR, MX1/MxA, and RIG-1. H2-Q6, histocompatibility 2, Q region locus 1 Gene, is involved in the presentation of foreign antigens to the immune system, which related to ovarian inflammation possibly like that in the uterine edema, which continued to adulthood in mouse model of this study. When estrogen treated mice entered into adulthood, there were still a lot of changes in the gene expression profiles. Crebbp, CREB binding protein Gene, acetylates histones, giving a specific tag for transcriptional activation; and in another role, it binds specifically to phosphorylate CREB and enhances its transcriptional activity toward cAMP-responsive genes. So in this study the blood insulin level drop can be explained by down-regulated expression of gene Crebbp and changes in its associated genes, according to its reported inhibitive effects on leptin and insulin-sensitizing [29]. C3 is a transglutaminase substrate, and indeed is covalently associated with the fibrin clot [30], so we hypothesized that individual follicles infiltrated with a large number of blood cells in adult mice with prepubertal estrogen treatment can be attributed to a decrease in C3 related blood coagulation.

In summary, estrogen treatment at the age of 3 weeks caused follicular synchronized development to arrest at early stage of the tertiary follicles, of which such follicles driven by exogenous estrogen do not respond to artificial superovulation, while the treatment had no obvious damages on the spontaneous follicles, for a similar number of oocytes were obtained by superovulation compared with control group. Moreover, the developmental potentials of the superovulated oocytes could be remained after estrogen stimulation, for normal offspring were produced from them using the artificial reproductive technology. On the other hand, although estrogen stimulation didn’t cause adult to appear typical symptoms of PCOS, the adult mouse ovarian tissues remained molecular changes related to inflammation.

## Supporting Information

S1 Table Reproductive outcome of superovulated oocytes derived from estrogen stimulated mice. (DOCX)

S2 Table Expression changes in the associated genes of mice after estrogen treatment from cDNA profiles. (DOCX)

S3 Table RT-PCR confirmation on expression changes in the associated genes of mice after estrogen treatment. (DOCX)

S4 Table Real time quantitative RT-PCR primer list. (DOCX)

S5 Table Gene lists from biological information analysis with significantly different expression caused by estrogen treatment. (DOCX)
