# Supplementary material for: Evaluation of Follicular Synchronization Caused by Estrogen Administration and Its Reproductive Outcome
Source: PLoS One. 2015 May 26;10(5):e0127595. doi: 10.1371/journal.pone.0127595 (PMC4444187; doi:10.1371/journal.pone.0127595)
Supplement: S1 Table — (DOCX) [file pone.0127595.s001.docx]

**S1 Table. Reproductive outcome of superovulated oocytes derived from estrogen stimulated mice.**

| Embryos from | Transfer site | Embryo stage | Recipient number | Embryo number | Offsprings |
| --- | --- | --- | --- | --- | --- |
| Young mice  (4 weeks old) | Uterine horn | Blastula | 3 | 31 | 16  （9♀, 7♂） |
|  | Fallopian tube | two-cell | 4 | 52 | 3（2♀, 1♂） |
| Adult mice (12weeks old) | Fallopian tube | Two-cell | 8 | 76 | 8（3♀, 5♂） |

As shown in S1 table, after artificial superovulation, in vitro fertilization, and embryo culture, 2-cell embryos were transfer into oviducts, and blastocysts were transferred into uterine horns of the recipient pseudopregnant mice. Results showed that oocytes from estrogen treated mice are of normal developmental potential to produce normal offspring by artificial reproductive technology.
