# Supplementary material for: Evaluation of Follicular Synchronization Caused by Estrogen Administration and Its Reproductive Outcome
Source: PLoS One. 2015 May 26;10(5):e0127595. doi: 10.1371/journal.pone.0127595 (PMC4444187; doi:10.1371/journal.pone.0127595)
Supplement: S2 Table — (DOCX) [file pone.0127595.s002.docx]

**S2 Table. Expression changes in the associated genes of mice after estrogen treatment from cDNA profiles.**

| Young（cDNA chip) | | |  | Adult (sequencing) | | |
| --- | --- | --- | --- | --- | --- | --- |
| gene | E2/Con | p_value |  | gene | log2(E2/Con) | p_value |
| Ap1m2 | 2.78 | 0.00 |  | Amot | -13.62 | 0.01 |
| Ccnd1 | 1.82 | 0.00 |  | Ap2a1 | 12.23 | 0.00 |
| Cd3g | 0.24 | 0.04 |  | Arnt | 12.40 | 0.00 |
| Cd74 | 0.64 | 0.00 |  | Atf2 | 11.14 | 0.01 |
| Cdk5r1 | 2.08 | 0.00 |  | Atm | -2.32 | 0.00 |
| Chad | 220.23 | 0.00 |  | C3 | -1.64 | 0.00 |
| E2f5 | 55.91 | 0.01 |  | C4a | -1.73 | 0.01 |
| Flnb | 1.63 | 0.00 |  | Carm1 | -11.22 | 0.01 |
| Gata2 | 1.85 | 0.00 |  | Cbl | -4.12 | 0.00 |
| H2-Ab1 | 0.58 | 0.00 |  | Cdon | -15.16 | 0.00 |
| H2-K1 | 0.49 | 0.00 |  | Cep110 | -14.39 | 0.01 |
| H2-L | 0.85 | 0.00 |  | Cep57 | -11.79 | 0.01 |
| H2-Q6 | 0.17 | 0.00 |  | Cited1 | 2.01 | 0.00 |
| H3f3a | 0.43 | 0.01 |  | Crebbp | -2.19 | 0.00 |
| Isg15 | 3.07 | 0.00 |  | Ctnnb1 | -10.90 | 0.01 |
| Itga2 | 0.61 | 0.00 |  |  |  |  |
| Jdp2 | 2.54 | 0.00 |  |  |  |  |
| Lama3 | 2.20 | 0.00 |  |  |  |  |
| Msx1 | 4.08 | 0.00 |  |  |  |  |
| Oas2 | 1.89 | 0.00 |  |  |  |  |
| Pgr | 4.20 | 0.00 |  |  |  |  |
| Rmcs2 | 0.55 | 0.00 |  |  |  |  |

In S2 table listed genes with significant associations deduced by program String 9.1. The gene lists applied were of significantly differential expression scanned through the cDNA chip technology (young mouse model) or by cDNA sequencing technology (adult mouse model). According to the previous research, using the String 9.1 program, the possible functional associations of genes with significant changes in expression were drawn to probe for molecular signaling pathways in response to estrogen stimulation in ovaries. Associations between genes were shown as in the Fig3.
