# Supplementary material for: Evaluation of Follicular Synchronization Caused by Estrogen Administration and Its Reproductive Outcome
Source: PLoS One. 2015 May 26;10(5):e0127595. doi: 10.1371/journal.pone.0127595 (PMC4444187; doi:10.1371/journal.pone.0127595)
Supplement: S3 Table — (DOCX) [file pone.0127595.s003.docx]

**S3 Table. RT-PCR confirmation on expression changes in the associated genes of mice after estrogen treatment.**

| Young (qRT-PCR） | |  | Adult(qRT-PCR） | |  |
| --- | --- | --- | --- | --- | --- |
| gene | E2/Con |  | gene | E2/Con |  |
| Ap1m2 | 1.03±0.13 |  | Amot |  |  |
| Ccnd1 | 0.94±0.13 |  | Ap2a1 |  |  |
| Cd3g | 0.82±0.27 |  | Arnt |  |  |
| Cd74 |  |  | Atf2 |  |  |
| Cdk5r1 |  |  | Atm |  |  |
| Chad |  |  | C3 | 0.83±0.41 |  |
| E2f5 |  |  | C4a |  |  |
| Flnb |  |  | Carm1 | 0.85±0.11 |  |
| Gata2 |  |  | Cbl |  |  |
| H2-Ab1 | 0.77±0.22 |  | Cdon |  |  |
| H2-K1 | 0.90±0.36 |  | Cep110 |  |  |
| H2-L |  |  | Cep57 |  |  |
| H2-Q6 | 0.88±0.16 |  | Cited1 | 1.41±1.24 |  |
| H3f3a |  |  | Crebbp | 0.98±0.24 |  |
| Isg15 |  |  | Ctnnb1 | 0.73±0.35 |  |
| Itga2 |  |  |  |  |  |
| Jdp2 |  |  |  |  |  |
| Lama3 |  |  |  |  |  |
| Msx1 |  |  |  |  |  |
| Oas2 |  |  |  |  |  |
| Pgr | 1.18±0.40 |  |  |  |  |
| Rmcs2 | 0.62±0.16 |  |  |  |  |

RNA extraction, RT reaction, and real-time PCR were performed to confirm expression differences of associated gens induced from the results of cDNA profiling by String 9.1 program. Total RNA was extracted from ovaries using TRIzol (Invitrogen, Karlsruhe, Germany). 1~2µg of total RNA was reverse transcribed into cDNA for PCR detection, 20µl of RT reaction mixture was used for PCR, and all the kits of RT-PCR was purchased from Takara Bio Group. The procedure was outlined as follows. 6.5µl of RNA, 1μl of oligo (dT) (10μM), 1μl of dNTP-mix (2.5 mM) and diethylpyrocarbonate (DEPC)-treated water up to 10μl were mixed and incubated at 65°C for 5 min and then immediately placed on ice. Next, 4μl of Primescript Buffer, 1μl of Primescript Reverse Transcriptase and DEPC-treated water were added. cDNA synthesis was carried out at 30°C for 10 min, then 42°C for 60 min. The reaction was stopped by incubating at 72°C for 10 min. The samples were then stored at -20°C until real-time PCR was performed. Real-time PCRs were performed using ABI Prism 7900HT real-time PCR detector (Applied Biosystems by Life Technologies). The reaction mixture consisted of 1μM of each primer for targeted genes, 5μl SYB green PCR master mix and 2μl of the DEPC water, 2μl corresponding cDNA in a total reaction volume of 10μl. The cDNA was denatured at 95°C for 1 min. The templates as then amplified over 40 cycles of 15 s of melting at 95°C, 15 s at 63°C for annealing and 45 s at 72°C for extension. The threshold cycle (Ct) values were normalized to GAPDH levels. Relative mRNA levels were calculated using the 2 ^-ΔΔCt^ method, as cited in S3 Table. All the data were presented as mean ± sd.
