# Supplementary material for: Evaluation of Follicular Synchronization Caused by Estrogen Administration and Its Reproductive Outcome
Source: PLoS One. 2015 May 26;10(5):e0127595. doi: 10.1371/journal.pone.0127595 (PMC4444187; doi:10.1371/journal.pone.0127595)
Supplement: S4 Table — (DOCX) [file pone.0127595.s004.docx]

**S4 table Real time quantitative RT-PCR primer list**

| **genes** | **primers (5'to3')** | **product(bp)** |
| --- | --- | --- |
| Ap1m2-1-F1 | CCTACAGTCACCAATGCGGT |  |
| Ap1m2-1-R2 | TGCCTGAAAGGCCAGTAAGC | 232 |
| Ccnd-1-F1 | AAAATGCCAGAGGCGGATGA |  |
| Ccnd-1-R1 | GAAAGTGCGTTGTGCGGTAG | 199 |
| Cd3g-1-F1 | TTCAAGGCACTGTAGCCCAG |  |
| Cd3g-1-R1 | CCTCGAGGGTCTTTGGCATT | 199 |
| H2-Ab1-1-F1 | CAGGTGTGAGTCCTGGTGAC |  |
| H2-Ab1-1-R1 | GTCCCGTTGGTGAAGTAGCA | 185 |
| H2-K1-1-F2 | CCTGGTGACTGCCATTACCT |  |
| H2-K1-1-R2 | GAAGTAGCACTCGCCCATGA | 163 |
| Pgr-1-F1 | CCCAGCATGTCGTCTGAGAA |  |
| Pgr-1-R1 | TGGCGGGACCAGTTGAATTT | 202 |
| Rmcs2-1-F1 | ACGGTGTGCAGACACAACTA |  |
| Rmcs2-1-R1 | GCCATTCCGGAACCATCTGA | 177 |
| C3-1-F2 | CAGAGCTGGTTGTGGACCAT |  |
| C3-1-R2 | TTCGTTGCGCACTACAGAGT | 225 |
| Cbl-1-F2 | ATTCTCCATGGCCCCACAAG |  |
| Cbl-1-R2 | GGTGGTGGTGGAAGATCTCG | 186 |
| Cited1-3-F1 | GGCGAGCTGTCCGTAAACAA |  |
| Cited1-3-R1 | GTGCAGGCCTCGACATAGTT | 228 |
| Crebbp-1-F2 | TCTCTCCAACATCCAACGGC |  |
| Crebbp-1-R2 | GTCTGAGGCTGTGGAGTCAC | 191 |
| Ctnnb1-1-F1 | GTCAGTGCAGGAGGCCG |  |
| Ctnnb1-1-R1 | CTTGCCACTCAGGGAAGGAG | 233 |
| Gapdh-F1 | TGCAGTGGCAAAGTGGAGATTGTTG |  |
| Gapdh-R1 | GGTCTCGCTCCTGGAAGATGGTGAT | 175 |

Primer sequences and the sizes of the expected PCR products are presented in S4 Table. The specificity of the primers used in the study was confirmed by agarose gel electrophoresis and sequence analysis on the reaction products.
