# Supplementary material for: Evaluation of Follicular Synchronization Caused by Estrogen Administration and Its Reproductive Outcome
Source: PLoS One. 2015 May 26;10(5):e0127595. doi: 10.1371/journal.pone.0127595 (PMC4444187; doi:10.1371/journal.pone.0127595)
Supplement: S5 Table — (DOCX) [file pone.0127595.s005.docx]

**S5 Table. Gene lists from biological information analysis with significantly different expression caused by estrogen treatment.**

| **Young（cDNA chip)** |  |  | | **Adult（cDNA seq)** | |
| --- | --- | --- | --- | --- | --- |
| **Gene Symbol** | **E2.Ratio** |  | **Gene Symbol** | | **E2.Ratio** |
| Insl3 | 0.00 |  | Snord37 | | -31.80 |
| A230077I10Rik | 0.01 |  | Gm22571 | | -30.69 |
| Twistnb | 0.04 |  | Actb | | -30.41 |
| Jak3 | 0.08 |  | mmu-mir-7237 | | -30.32 |
| Prss22 | 0.11 |  | Ctsd | | -29.94 |
| BC010462 | 0.12 |  | Canx | | -29.91 |
| Cyp17a1 | 0.12 |  | Gm25206 | | -29.37 |
| Star | 0.12 |  | Ywhae | | -28.76 |
| Rhox8 | 0.13 |  | Bcat1 | | -28.61 |
| Cxcl9 | 0.14 |  | Uqcrc2 | | -28.57 |
| Hao3 | 0.17 |  | Hnrnpu | | -28.56 |
| H2-Q6 | 0.17 |  | Gm24366 | | -28.54 |
| Nkg7 | 0.18 |  | AC124170.1 | | -27.84 |
| 0610037M15Rik | 0.19 |  | n-R5s159 | | -26.78 |
| 9030625A04Rik | 0.20 |  | Arhgap29 | | -26.74 |
| Mucdhl | 0.24 |  | GNAS-AS1_4 | | -26.69 |
| Cd3g | 0.24 |  | Nfe2l1 | | -26.61 |
| G630023A01Rik | 0.25 |  | GNAS-AS1_5 | | -26.57 |
| 1700017I11Rik | 0.26 |  | Ighv7-4 | | -26.35 |
| Ms4a10 | 0.27 |  | Gm22642 | | -26.12 |
| 1700071K01Rik | 0.27 |  | Igkv4-57-1 | | -25.96 |
| Fads2 | 0.27 |  | Igkv4-61 | | -25.62 |
| Sct | 0.29 |  | Ighv2-5 | | -25.35 |
| Hsd3b1 | 0.30 |  | Ighv1-4 | | -25.17 |
| 2500002A22Rik | 0.30 |  | 1700024G13Rik | | -25.05 |
| Dpep1 | 0.31 |  | Zcchc14 | | -25.03 |
| Tcrb-V13 | 0.31 |  | Igkv4-59 | | -25.01 |
| D030068E18Rik | 0.32 |  | 2310007L24Rik | | -24.98 |
| Ipas | 0.32 |  | Pifo | | -24.82 |
| Aard | 0.32 |  | Igkv3-10 | | -24.81 |
| LOC381399 | 0.33 |  | Gm21655 | | -24.69 |
| Orm1 | 0.34 |  | 1700016K19Rik | | -24.49 |
| 4921530G04Rik | 0.34 |  | Gm22314 | | -24.25 |
| 2210413P12Rik | 0.35 |  | Irf8 | | -24.18 |
| Ptx3 | 0.35 |  | Mpo | | -24.08 |
| A430077H15Rik | 0.36 |  | Afg3l1 | | -24.03 |
| Mro | 0.36 |  | Def8 | | -24.01 |
| Sectm1 | 0.37 |  | Zfpm1 | | -23.99 |
| Fabp3 | 0.37 |  | Zfpm1 | | -23.87 |
| 1700024G10Rik | 0.38 |  | Wfdc15b | | -23.81 |
| 2410131K14Rik | 0.38 |  | Def8 | | -23.81 |
| A330017A19Rik | 0.38 |  | 2010007H06Rik | | -23.81 |
| Ccng2 | 0.39 |  | Vps9d1 | | -23.78 |
| Ccl5 | 0.39 |  | Banp | | -23.73 |
| Ush1g | 0.39 |  | Zc3h18 | | -23.64 |
| Scd1 | 0.39 |  | Irf8 | | -23.64 |
| Scrn2 | 0.40 |  | Dos | | -23.56 |
| mKIAA0282 | 0.40 |  | Zfp474 | | -23.53 |
| H1foo | 0.40 |  | H2-M2 | | -23.39 |
| Olfr873 | 0.41 |  | Mup10 | | -23.38 |
| Gch1 | 0.42 |  | Gm8540 | | -23.35 |
| Inhba | 0.43 |  | Stxbp3b | | -23.33 |
| Prss35 | 0.43 |  | Vps9d1 | | -23.33 |
| 2610511O17Rik | 0.43 |  | Plekha1 | | -23.27 |
| 5430417J04Rik | 0.43 |  | Skp1a | | -23.24 |
| D130052N13Rik | 0.43 |  | Gm12105 | | -23.23 |
| 1110025G12Rik | 0.43 |  | Rps12-ps9 | | -23.04 |
| 9430068D06Rik | 0.43 |  | 4932418E24Rik | | -23.03 |
| Kcnk3 | 0.44 |  | Sox3 | | -22.92 |
| Gsta2 | 0.44 |  | 2610528J11Rik | | -22.87 |
| LOC385461 | 0.44 |  | Mthfsd | | -22.85 |
| Chst3 | 0.44 |  | H2-M2 | | -22.75 |
| BC011210 | 0.44 |  | E030019B06Rik | | -22.73 |
| Stk32a | 0.44 |  | Fam168a | | -16.04 |
| E030045D18Rik | 0.44 |  | Slfn4 | | -15.95 |
| MGC117731 | 0.45 |  | Wdhd1 | | -15.37 |
| Gm1006 | 0.46 |  | Dennd4b | | -15.31 |
| Pla2g1b | 0.46 |  | Cyfip1 | | -15.30 |
| Foxa3 | 0.47 |  | Cdon | | -15.16 |
| D030035F05Rik | 0.47 |  | Skil | | -14.93 |
| H2-K1 | 0.49 |  | Golgb1 | | -14.49 |
| Acsbg1 | 0.49 |  | Abi1 | | -14.44 |
| Rbbp9 | 0.49 |  | Cep110 | | -14.39 |
| 6030408B16Rik | 0.49 |  | Trp53bp1 | | -14.33 |
| Doc2b | 0.49 |  | Dock7 | | -14.32 |
| Aldh1b1 | 0.50 |  | Wdr37 | | -14.28 |
| Cyp19a1 | 0.51 |  | Gulp1 | | -14.21 |
| Niban | 0.51 |  | Zfp952 | | -14.04 |
| Dlk1 | 0.51 |  | Ano1 | | -14.03 |
| scl0001849.1_2273 | 0.51 |  | Vps18 | | -14.01 |
| Heyl | 0.52 |  | Ptp4a3 | | -14.01 |
| Npm2 | 0.52 |  | Eps8 | | -13.99 |
| Cldn11 | 0.53 |  | Zmiz2 | | -13.97 |
| Scara5 | 0.53 |  | Zfp58 | | -13.95 |
| Pgrmc1 | 0.53 |  | Pdp1 | | -13.88 |
| Wnt6 | 0.53 |  | Rbm39 | | -13.87 |
| LOC384281 | 0.55 |  | Ankra2 | | -13.87 |
| 8030402P03Rik | 0.55 |  | Ggnbp2 | | -13.85 |
| 9430043O10Rik | 0.55 |  | Zfp275 | | -13.81 |
| Slc18a2 | 0.55 |  | Stk38l | | -13.75 |
| Gpha2 | 0.55 |  | Slc39a3 | | -13.72 |
| Atp10a | 0.55 |  | Igsf9 | | -13.70 |
| Phactr4 | 0.55 |  | Jak2 | | -13.70 |
| H2-Eb1 | 0.55 |  | Tbc1d4 | | -13.68 |
| Fads1 | 0.56 |  | Cyth1 | | -13.67 |
| 1110020P09Rik | 0.56 |  | Oxr1 | | -13.65 |
| Mod1 | 0.56 |  | Amot | | -13.62 |
| 0610030P10Rik | 0.57 |  | Mink1 | | -13.61 |
| E330017A01Rik | 0.57 |  | Rev3l | | -13.57 |
| D230034L24Rik | 0.57 |  | Smtn | | -13.51 |
| Pak3 | 0.57 |  | Ehbp1 | | -13.51 |
| LOC386473 | 0.58 |  | Wasf1 | | -13.50 |
| LOC331139 | 0.58 |  | Nup210 | | -13.48 |
| 1110012J17Rik | 0.58 |  | Syt7 | | -13.46 |
| Rgs2 | 0.58 |  | Mum1l1 | | -13.46 |
| Prkag2 | 0.58 |  | Nrep | | -13.44 |
| H2-Ab1 | 0.58 |  | Ttc7 | | -13.43 |
| Ssb4 | 0.59 |  | Tmem164 | | -13.42 |
| Tcf21 | 0.59 |  | Tle4 | | -13.36 |
| Ris2 | 0.59 |  | Sun1 | | -13.31 |
| Kif2c | 0.59 |  | Rhobtb1 | | -13.23 |
| Hist1h2ak | 0.59 |  | Zfp248 | | -13.15 |
| LOC231423 | 0.60 |  | Cse1l | | -13.14 |
| LOC382215 | 0.60 |  | Sp3 | | -13.11 |
| Sytl4 | 0.60 |  | Mtus1 | | -13.11 |
| Arrdc3 | 0.61 |  | Smc2 | | -13.07 |
| Itga2 | 0.61 |  | Smox | | -13.05 |
| Hsp105 | 0.61 |  | Cldn12 | | -13.04 |
| 4432411E13Rik | 0.61 |  | Vps54 | | -13.01 |
| 9930105H17Rik | 0.61 |  | Tctn1 | | -13.00 |
| BC099439 | 0.61 |  | Rtel1 | | -12.93 |
| Hmgcs1 | 0.61 |  | Chtf8 | | -12.93 |
| 4930583H14Rik | 0.61 |  | Abhd13 | | -12.92 |
| Prkar2b | 0.62 |  | Synj2 | | -12.90 |
| Slc7a5 | 0.62 |  | Mier3 | | -12.87 |
| LOC382105 | 0.62 |  | Lyar | | -12.87 |
| Hoxd9 | 0.62 |  | Prpsap2 | | -12.87 |
| 0610010E21Rik | 0.63 |  | Cbfb | | -12.85 |
| A430023D23Rik | 0.63 |  | Nat9 | | -12.84 |
| 2210415K03Rik | 0.63 |  | Myc | | -12.81 |
| Map4k3 | 0.63 |  | Wdr62 | | -12.79 |
| Ii | 0.64 |  | Prickle1 | | -12.78 |
| Defb19 | 0.64 |  | Zzz3 | | -12.68 |
| 5430426F23Rik | 0.64 |  | Cyb561d1 | | -12.61 |
| 2900019M05Rik | 0.65 |  | Abhd3 | | -12.60 |
| 3830612M24 | 0.65 |  | Ppil2 | | -12.57 |
| 6720475J19Rik | 0.66 |  | Vps39 | | -12.56 |
| LOC228862 | 0.67 |  | Spen | | -12.53 |
| 9430052C07Rik | 0.67 |  | Usp20 | | -12.52 |
| Pace4 | 0.68 |  | Hdac6 | | -12.49 |
| Ercc5 | 0.73 |  | Mybpc3 | | -12.49 |
| Rhobtb1 | 0.76 |  | Fam161a | | -12.32 |
| H2-D1 | 0.85 |  | Lrrc8d | | -12.31 |
| H2-Q5 | 0.91 |  | Galnt11 | | -12.29 |
| Hnrpa0 | 1.04 |  | Tbc1d7 | | -12.26 |
| Epb4.1l2 | 1.45 |  | Gga3 | | -12.26 |
| 1700007K13Rik | 1.53 |  | Echdc2 | | -12.24 |
| D630040I23Rik | 1.56 |  | Dtnb | | -12.21 |
| 1810029C22Rik | 1.56 |  | Gpcpd1 | | -12.20 |
| 1200016E24Rik | 1.58 |  | Mapkap1 | | -12.15 |
| Flnb | 1.63 |  | Pnkd | | -12.14 |
| Hspb8 | 1.65 |  | Diap1 | | -12.14 |
| 6430559E15Rik | 1.66 |  | Txk | | -12.12 |
| Lgals3 | 1.67 |  | Pja2 | | -12.10 |
| Hbb-b1 | 1.67 |  | Rcor3 | | -12.08 |
| Cmas | 1.67 |  | Src | | -12.08 |
| Tsga2 | 1.68 |  | Tia1 | | -12.03 |
| Lox | 1.69 |  | Runx1 | | -11.99 |
| Pfc | 1.69 |  | Sqstm1 | | -11.98 |
| Myl9 | 1.69 |  | Gabpb1 | | -11.97 |
| Plekhb2 | 1.70 |  | Bmi1 | | -11.92 |
| Msr2 | 1.70 |  | Unkl | | -11.91 |
| Mrpl27 | 1.71 |  | Myo18a | | -11.86 |
| 4933428D01Rik | 1.71 |  | Setdb1 | | -11.86 |
| Zfp36 | 1.73 |  | Cep112 | | -11.86 |
| D1Ertd471e | 1.73 |  | Slu7 | | -11.79 |
| Fcgr3 | 1.74 |  | Shoc2 | | -11.79 |
| Ifitm3 | 1.74 |  | Cep57 | | -11.79 |
| 1110017D15Rik | 1.75 |  | Uhmk1 | | -11.69 |
| 1200006F02Rik | 1.76 |  | Dcaf11 | | -11.62 |
| LOC382087 | 1.76 |  | Ubtf | | -11.60 |
| 9330140K16Rik | 1.77 |  | Birc6 | | -11.52 |
| Msrb2 | 1.77 |  | Tdrd3 | | -11.45 |
| 1110061N23Rik | 1.78 |  | Patz1 | | -11.44 |
| Cyb561 | 1.78 |  | Daxx | | -11.37 |
| Lrrc34 | 1.78 |  | Plekhg5 | | -11.36 |
| scl0003519.1_175 | 1.78 |  | Dmpk | | -11.29 |
| LOC383411 | 1.79 |  | Ino80d | | -11.28 |
| 1300002F13Rik | 1.79 |  | Rnf123 | | -11.27 |
| LOC381738 | 1.79 |  | Limd2 | | -11.26 |
| Mycbpap | 1.79 |  | Carm1 | | -11.22 |
| 1700001C02Rik | 1.80 |  | Tbc1d31 | | -11.15 |
| Gsto1 | 1.80 |  | Ctnnb1 | | -10.90 |
| 4930455F23Rik | 1.80 |  | Trim47 | | -10.89 |
| Plvap | 1.80 |  | Dhx30 | | -10.85 |
| Napsa | 1.80 |  | Rngtt | | -10.33 |
| Ppil6 | 1.81 |  | BC048546 | | -5.26 |
| 1600029I14Rik | 1.81 |  | Fosb | | -4.76 |
| Gpx1 | 1.82 |  | S100a9 | | -4.63 |
| Gadd45a | 1.82 |  | Zfp871 | | -4.60 |
| Ccnd1 | 1.82 |  | S100a8 | | -4.42 |
| Apobec1 | 1.82 |  | Inhba | | -4.22 |
| Nme5 | 1.83 |  | Cbl | | -4.12 |
| Laptm5 | 1.83 |  | Edn2 | | -3.97 |
| Wfs1 | 1.83 |  | Gm20400 | | -3.80 |
| 1700120B06Rik | 1.84 |  | Lmbrd2 | | -3.78 |
| Smpdl3a | 1.84 |  | Gm5862 | | -3.58 |
| Gata2 | 1.85 |  | Lcor | | -3.55 |
| 2900093B09Rik | 1.86 |  | Dusp4 | | -3.51 |
| Hoxb7 | 1.86 |  | Gatad2b | | -3.46 |
| Sca1 | 1.86 |  | Gm21847 | | -3.44 |
| Stard5 | 1.86 |  | Wdfy2 | | -3.42 |
| C230072F16Rik | 1.86 |  | Aff2 | | -3.41 |
| Temt | 1.87 |  | Gm12505 | | -3.29 |
| 1700027A23Rik | 1.88 |  | Arid5b | | -3.29 |
| 1700003M02Rik | 1.88 |  | Phc3 | | -3.24 |
| Oas2 | 1.89 |  | Bmpr2 | | -3.11 |
| Bcat1 | 1.89 |  | Adamts12 | | -3.06 |
| Des | 1.89 |  | Slc8a1 | | -3.05 |
| 9630019K15Rik | 1.91 |  | Atad2b | | -3.04 |
| 1700127F16Rik | 1.91 |  | Mdm4 | | -3.04 |
| Spdef | 1.91 |  | Pdpr | | -3.03 |
| Cd9 | 1.92 |  | Cgnl1 | | -2.99 |
| Dncl2b | 1.92 |  | Vash1 | | -2.98 |
| 9630007E23Rik | 1.93 |  | Tnks | | -2.97 |
| Dcxr | 1.93 |  | Itga2 | | -2.92 |
| Krtcap3 | 1.93 |  | Lnpep | | -2.88 |
| D5Ertd593e | 1.94 |  | Srcap | | -2.87 |
| Biklk | 1.94 |  | Gm12840 | | -2.84 |
| 1700028N11Rik | 1.94 |  | Klhl28 | | -2.81 |
| 1190003J15Rik | 1.95 |  | Ighg2b | | -2.81 |
| Adamts4 | 1.95 |  | Tnfaip6 | | -2.80 |
| 1110049B09Rik | 1.95 |  | Aak1 | | -2.77 |
| E130201N16Rik | 1.95 |  | Ptch1 | | -2.75 |
| 1700024G13Rik | 1.96 |  | Zfp445 | | -2.74 |
| Lgals7 | 1.96 |  | Kif13b | | -2.74 |
| 2610028H24Rik | 1.97 |  | Anxa8 | | -2.74 |
| Cyp7b1 | 1.98 |  | Tmem26 | | -2.72 |
| Stard10 | 1.98 |  | Gm7292 | | -2.70 |
| Grb7 | 1.99 |  | Mast4 | | -2.68 |
| Rshl3 | 1.99 |  | Speer7-ps1 | | -2.63 |
| Ccl8 | 1.99 |  | Pde3a | | -2.60 |
| 2310061N23Rik | 1.99 |  | Gm5067 | | -2.57 |
| D4Ertd432e | 2.00 |  | Cdsn | | -2.57 |
| 3100002J23Rik | 2.01 |  | Tnrc18 | | -2.56 |
| Sema4a | 2.01 |  | Pm20d2 | | -2.56 |
| Pacrg | 2.01 |  | Ccdc88c | | -2.51 |
| Fndc7 | 2.02 |  | Btbd7 | | -2.50 |
| Cldn3 | 2.02 |  | Errfi1 | | -2.50 |
| 1700008D07Rik | 2.02 |  | Lyst | | -2.49 |
| 1110008I14Rik | 2.02 |  | Irs2 | | -2.40 |
| Hip1r | 2.03 |  | Klf3 | | -2.40 |
| Sepm | 2.03 |  | Klhl11 | | -2.38 |
| Krt1-12 | 2.03 |  | Vps9d1 | | -2.32 |
| 4933417K04Rik | 2.05 |  | Atm | | -2.32 |
| Ovgp1 | 2.05 |  | Sprtn | | -2.27 |
| LOC381229 | 2.06 |  | Lrrc8b | | -2.25 |
| 1700007G11Rik | 2.07 |  | Palld | | -2.23 |
| Cdk5r1 | 2.08 |  | Mical3 | | -2.22 |
| S100a6 | 2.08 |  | Rnf152 | | -2.22 |
| Cdh16 | 2.08 |  | Abca6 | | -2.21 |
| P2ry14 | 2.09 |  | Crebbp | | -2.19 |
| 1810015C04Rik | 2.09 |  | Alg10b | | -2.19 |
| Lgals2 | 2.09 |  | 1600029D21Rik | | -2.18 |
| Cyp2e1 | 2.10 |  | Kl | | -2.18 |
| BC038167 | 2.11 |  | Sema3d | | -2.13 |
| Efcab4a | 2.11 |  | 2410066E13Rik | | -2.10 |
| E030019B06Rik | 2.12 |  | Stc1 | | -2.10 |
| Mgst2 | 2.12 |  | Adamts6 | | -2.07 |
| LOC381000 | 2.13 |  | Eif4ebp2 | | -2.04 |
| 4932425I24Rik | 2.14 |  | Soga1 | | -2.03 |
| Trf | 2.14 |  | Masp1 | | -2.02 |
| Fzd6 | 2.14 |  | Zc3h12c | | -1.99 |
| LOC385755 | 2.14 |  | Plxna4 | | -1.97 |
| AW212394 | 2.15 |  | Gm26809 | | -1.97 |
| Igfbp6 | 2.15 |  | Uba6 | | -1.97 |
| Timp4 | 2.16 |  | Pkhd1l1 | | -1.95 |
| BC036564 | 2.16 |  | Atf3 | | -1.95 |
| Hoxb2 | 2.16 |  | Vps13c | | -1.93 |
| Ak1 | 2.17 |  | Ndst1 | | -1.93 |
| Sox9 | 2.17 |  | Arhgap5 | | -1.92 |
| B230312L03Rik | 2.18 |  | Gpam | | -1.88 |
| Krt1-23 | 2.18 |  | Megf10 | | -1.86 |
| Itpr3 | 2.18 |  | 1110002E22Rik | | -1.85 |
| Lama3 | 2.20 |  | Mdn1 | | -1.81 |
| LOC386405 | 2.21 |  | Sntb2 | | -1.80 |
| 1700009P17Rik | 2.24 |  | Sesn3 | | -1.78 |
| Gm867 | 2.24 |  | Fat1 | | -1.76 |
| 2310040A07Rik | 2.25 |  | Btg2 | | -1.76 |
| Mdh1b | 2.27 |  | Vps13a | | -1.75 |
| Prss8 | 2.27 |  | Ppp1r12a | | -1.74 |
| 4932443I19Rik | 2.27 |  | C4b | | -1.73 |
| Akr1c19 | 2.28 |  | Dock4 | | -1.68 |
| 1500031H04Rik | 2.28 |  | C3 | | -1.64 |
| Fgf1 | 2.29 |  | Atf7ip | | -1.63 |
| LOC386091 | 2.29 |  | Zfp609 | | -1.60 |
| AU021034 | 2.29 |  | Pds5a | | -1.59 |
| 4833436C18Rik | 2.29 |  | Slc7a1 | | -1.57 |
| Slco2a1 | 2.30 |  | Nrcam | | -1.50 |
| 4922503N01Rik | 2.30 |  | Smg1 | | -1.49 |
| Cmtm8 | 2.31 |  | Eef1a1 | | -1.29 |
| LOC331595 | 2.32 |  | Gm15775 | | -1.28 |
| Fxyd4 | 2.33 |  | Spg7 | | -1.12 |
| Slamf9 | 2.34 |  | Gm16378 | | 1.11 |
| Igh-VJ558 | 2.34 |  | Cbfa2t3 | | 1.13 |
| 1700021K14Rik | 2.35 |  | Abcb1b | | 1.83 |
| BC007180 | 2.36 |  | Cited1 | | 2.01 |
| Wisp2 | 2.37 |  | 4930486L24Rik | | 2.64 |
| BC024997 | 2.37 |  | Bpifb6 | | 2.83 |
| scl0003300.1_40 | 2.38 |  | Gyltl1b | | 11.05 |
| BC018371 | 2.39 |  | Als2cl | | 11.07 |
| BC054059 | 2.39 |  | Atf2 | | 11.14 |
| AI118078 | 2.39 |  | Acin1 | | 11.15 |
| 3300002A11Rik | 2.39 |  | Acad11 | | 11.26 |
| Serpina3n | 2.40 |  | Dgkq | | 11.27 |
| Ramp3 | 2.40 |  | Gga3 | | 11.28 |
| AW228700 | 2.40 |  | Phldb1 | | 11.43 |
| 1700066F09Rik | 2.41 |  | Cacna1h | | 11.60 |
| Acox2 | 2.41 |  | C1qtnf1 | | 11.61 |
| Cldn15 | 2.41 |  | Ndufaf1 | | 11.64 |
| AI427122 | 2.42 |  | Suv420h2 | | 11.69 |
| Wfdc15 | 2.42 |  | Pld2 | | 11.82 |
| 4932408C11Rik | 2.42 |  | Abhd12 | | 11.83 |
| Tacstd2 | 2.43 |  | Egr2 | | 11.98 |
| Mgl2 | 2.43 |  | Ncstn | | 12.01 |
| Ugt1a9 | 2.47 |  | Pfkfb3 | | 12.02 |
| BC025206 | 2.47 |  | Ttc23 | | 12.06 |
| Alas2 | 2.47 |  | Cgnl1 | | 12.14 |
| Mustn1 | 2.48 |  | Tgif1 | | 12.23 |
| Treml1 | 2.48 |  | Ap2a1 | | 12.23 |
| 6330530A05Rik | 2.50 |  | Sgk3 | | 12.29 |
| Car12 | 2.50 |  | Nme6 | | 12.31 |
| Pscd4 | 2.51 |  | Dgcr2 | | 12.33 |
| Stc2 | 2.51 |  | Sgsh | | 12.38 |
| 2310007L24Rik | 2.52 |  | Arnt | | 12.40 |
| Slc9a3r1 | 2.53 |  | Nadk2 | | 12.45 |
| Prrx2 | 2.53 |  | Ercc2 | | 12.54 |
| Gmds | 2.54 |  | Papd5 | | 12.77 |
| Jundm2 | 2.54 |  | Itga10 | | 12.79 |
| Ccl12 | 2.54 |  | Ift20 | | 12.79 |
| BC028975 | 2.56 |  | Ggnbp2 | | 12.87 |
| Dmkn | 2.57 |  | Trip4 | | 13.06 |
| BC021891 | 2.57 |  | Rab22a | | 13.09 |
| Aqp8 | 2.57 |  | Cct3 | | 13.13 |
| Cfd | 2.57 |  | Acer2 | | 13.46 |
| Ugt1a10 | 2.58 |  | Sspn | | 13.56 |
| Igfbp2 | 2.58 |  | Zfp184 | | 13.69 |
| Krt1-18 | 2.58 |  | Vps33b | | 13.70 |
| Rgs4 | 2.62 |  | 4930506M07Rik | | 13.71 |
| Cxcl4 | 2.63 |  | Atp10d | | 13.84 |
| A830030H10Rik | 2.64 |  | Stag2 | | 13.93 |
| Ltf | 2.64 |  | Nlrp14 | | 13.98 |
| Fpr-rs2 | 2.64 |  | Ap1s2 | | 14.15 |
| 5730402C02Rik | 2.64 |  | Pnpla2 | | 14.32 |
| Ak7 | 2.64 |  | Ncoa5 | | 14.35 |
| Spon2 | 2.64 |  | Cpsf4 | | 14.40 |
| Tex2 | 2.66 |  | Pomgnt1 | | 14.44 |
| Muc1 | 2.66 |  | Ino80c | | 14.62 |
| Pitpnm3 | 2.68 |  | Kcnk2 | | 14.63 |
| Aox3 | 2.71 |  | Esco1 | | 14.64 |
| Slp | 2.71 |  | Gm17786 | | 22.79 |
| Cited4 | 2.72 |  | Hsp90ab1 | | 22.99 |
| LOC207939 | 2.73 |  | 1700007K09Rik | | 23.29 |
| Gng13 | 2.74 |  | Gm24239 | | 23.60 |
| Atad4 | 2.75 |  | Sult5a1 | | 23.85 |
| Tm4sf5 | 2.76 |  | Gm13321 | | 24.30 |
| Galntl4 | 2.77 |  | Klhdc4 | | 24.68 |
| A330021E22Rik | 2.78 |  | Scarna13 | | 25.60 |
| Gpx2 | 2.78 |  | Snord15a | | 26.22 |
| Speer4c | 2.78 |  | Gm25428 | | 27.57 |
| Ap1m2 | 2.78 |  | Ldhb | | 29.41 |
| F13a1 | 2.80 |  | Mir704 | | 29.67 |
| Folr2 | 2.81 |  | Gm23136 | | 29.90 |
| LOC381625 | 2.81 |  | Mir677 | | 30.05 |
| Tcfcp2l1 | 2.83 |  | Gm22455 | | 31.71 |
| LOC381928 | 2.84 |  |  | |  |
| 1700026L06Rik | 2.87 |  |  | |  |
| Reprimo | 2.89 |  |  | |  |
| Ces3 | 2.90 |  |  | |  |
| Pkp1 | 2.90 |  |  | |  |
| Ier3 | 2.91 |  |  | |  |
| A730032D07Rik | 2.93 |  |  | |  |
| E330013P04Rik | 2.94 |  |  | |  |
| Tm4sf3 | 2.95 |  |  | |  |
| C3 | 2.96 |  |  | |  |
| Cbs | 2.97 |  |  | |  |
| 6430704N06 | 2.98 |  |  | |  |
| LOC380842 | 2.98 |  |  | |  |
| Pcdh17 | 2.98 |  |  | |  |
| Alox5ap | 3.02 |  |  | |  |
| Gm644 | 3.02 |  |  | |  |
| Chchd4 | 3.05 |  |  | |  |
| Vmd2 | 3.06 |  |  | |  |
| G1p2 | 3.07 |  |  | |  |
| LOC100034251 | 3.08 |  |  | |  |
| Sn | 3.09 |  |  | |  |
| Rapgefl1 | 3.09 |  |  | |  |
| Cfi | 3.10 |  |  | |  |
| Cldn2 | 3.11 |  |  | |  |
| 1700019F09Rik | 3.11 |  |  | |  |
| Scin | 3.12 |  |  | |  |
| D3Ertd751e | 3.12 |  |  | |  |
| LOC380766 | 3.14 |  |  | |  |
| 2210401J11Rik | 3.15 |  |  | |  |
| Krt7 | 3.20 |  |  | |  |
| Ttc21a | 3.26 |  |  | |  |
| LOC226866 | 3.26 |  |  | |  |
| 1700019N12Rik | 3.28 |  |  | |  |
| Rab25 | 3.28 |  |  | |  |
| Upk1a | 3.34 |  |  | |  |
| 5031410I06Rik | 3.34 |  |  | |  |
| C230026C11 | 3.35 |  |  | |  |
| D430011E20Rik | 3.35 |  |  | |  |
| LOC381150 | 3.36 |  |  | |  |
| 3110035E14Rik | 3.38 |  |  | |  |
| Defb14 | 3.38 |  |  | |  |
| Fcna | 3.39 |  |  | |  |
| 2310006E12Rik | 3.40 |  |  | |  |
| Rtn1 | 3.40 |  |  | |  |
| Slc15a2 | 3.40 |  |  | |  |
| Dcpp | 3.41 |  |  | |  |
| Slc2a3 | 3.42 |  |  | |  |
| Krt1-19 | 3.42 |  |  | |  |
| Tmprss2 | 3.46 |  |  | |  |
| Ly6h | 3.47 |  |  | |  |
| Moxd1 | 3.48 |  |  | |  |
| BC004853 | 3.48 |  |  | |  |
| Exoc7 | 3.51 |  |  | |  |
| Vpreb3 | 3.51 |  |  | |  |
| Bspry | 3.53 |  |  | |  |
| 1200013B22Rik | 3.55 |  |  | |  |
| 0610037B23Rik | 3.56 |  |  | |  |
| Car13 | 3.59 |  |  | |  |
| Cbr2 | 3.61 |  |  | |  |
| Igl-V1 | 3.61 |  |  | |  |
| LOC218617 | 3.62 |  |  | |  |
| 1700086L19Rik | 3.64 |  |  | |  |
| Ccl9 | 3.68 |  |  | |  |
| Cd14 | 3.73 |  |  | |  |
| Cda | 3.73 |  |  | |  |
| Cyp26a1 | 3.82 |  |  | |  |
| Tmod1 | 3.87 |  |  | |  |
| Lrg1 | 3.90 |  |  | |  |
| scl0003073.1_164 | 3.91 |  |  | |  |
| Dfy | 3.94 |  |  | |  |
| Ddo | 3.96 |  |  | |  |
| Anxa8 | 3.97 |  |  | |  |
| Serpinb11 | 4.00 |  |  | |  |
| Msx1 | 4.08 |  |  | |  |
| Igh-6 | 4.09 |  |  | |  |
| Spint1 | 4.11 |  |  | |  |
| Alox12e | 4.19 |  |  | |  |
| Pgr | 4.20 |  |  | |  |
| Wnt7b | 4.22 |  |  | |  |
| Ifnt1 | 4.25 |  |  | |  |
| Dcpp1 | 4.27 |  |  | |  |
| Slc25a18 | 4.29 |  |  | |  |
| BC005655 | 4.32 |  |  | |  |
| Cidea | 4.33 |  |  | |  |
| 4632417N05Rik | 4.36 |  |  | |  |
| Cldn10 | 4.41 |  |  | |  |
| AW123240 | 4.43 |  |  | |  |
| Hsd11b1 | 4.43 |  |  | |  |
| Smpd3 | 4.44 |  |  | |  |
| Gria1 | 4.48 |  |  | |  |
| Ptgds2 | 4.62 |  |  | |  |
| Elf3 | 4.65 |  |  | |  |
| Slc6a2 | 4.72 |  |  | |  |
| Abp1 | 4.72 |  |  | |  |
| Cldn23 | 4.82 |  |  | |  |
| 1110018K11Rik | 4.83 |  |  | |  |
| Msln | 4.87 |  |  | |  |
| C130060K24Rik | 4.87 |  |  | |  |
| LOC381736 | 4.89 |  |  | |  |
| Ear1 | 4.89 |  |  | |  |
| Rab6b | 4.90 |  |  | |  |
| Ear2 | 4.90 |  |  | |  |
| 1810008K03Rik | 4.94 |  |  | |  |
| 4833409N03Rik | 5.05 |  |  | |  |
| Slc14a1 | 5.07 |  |  | |  |
| 2610203C22Rik | 5.33 |  |  | |  |
| 1600029D21Rik | 5.37 |  |  | |  |
| BC024561 | 5.62 |  |  | |  |
| 2310042N02Rik | 5.67 |  |  | |  |
| 2010300G19Rik | 5.86 |  |  | |  |
| Ccl11 | 5.89 |  |  | |  |
| Ear4 | 5.91 |  |  | |  |
| Il20rb | 5.96 |  |  | |  |
| LOC620807 | 6.10 |  |  | |  |
| A330049M08Rik | 6.28 |  |  | |  |
| Pglyrp1 | 6.32 |  |  | |  |
| Dgkg | 6.33 |  |  | |  |
| Eraf | 6.36 |  |  | |  |
| Eppk1 | 6.38 |  |  | |  |
| 4933428F06Rik | 6.53 |  |  | |  |
| Gal3st1 | 6.89 |  |  | |  |
| Trem2 | 6.95 |  |  | |  |
| Krt15 | 7.18 |  |  | |  |
| Nalp6 | 7.54 |  |  | |  |
| C230066H16Rik | 7.58 |  |  | |  |
| BC048546 | 7.65 |  |  | |  |
| Hp | 7.69 |  |  | |  |
| Il1b | 7.77 |  |  | |  |
| Sftpd | 7.87 |  |  | |  |
| Klk21 | 7.92 |  |  | |  |
| LOC381914 | 7.94 |  |  | |  |
| LOC232400 | 8.02 |  |  | |  |
| Klk1b27 | 8.22 |  |  | |  |
| Crlf1 | 8.43 |  |  | |  |
| Hgfac | 8.49 |  |  | |  |
| Padi1 | 8.61 |  |  | |  |
| S100a8 | 8.79 |  |  | |  |
| Cldn22 | 8.91 |  |  | |  |
| Klk24 | 9.49 |  |  | |  |
| Expi | 10.63 |  |  | |  |
| S100a9 | 10.66 |  |  | |  |
| Ghrh | 11.31 |  |  | |  |
| E030025L21Rik | 11.42 |  |  | |  |
| Klk5 | 11.98 |  |  | |  |
| Lcn2 | 12.03 |  |  | |  |
| scl0002073.1_13 | 12.61 |  |  | |  |
| Pkp3 | 12.85 |  |  | |  |
| 4933427D14Rik | 13.10 |  |  | |  |
| Edn2 | 13.50 |  |  | |  |
| A730013H24Rik | 13.86 |  |  | |  |
| Ly6f | 15.44 |  |  | |  |
| IGKV9-128_AJ231245  _Ig_kappa_variable_9-128_15 | 15.88 |  |  | |  |
| D630002J15Rik | 16.56 |  |  | |  |
| Ryd5 | 16.72 |  |  | |  |
| Isp2 | 17.40 |  |  | |  |
| Cdsn | 17.76 |  |  | |  |
| Arg1 | 20.38 |  |  | |  |
| Cuzd1 | 22.07 |  |  | |  |
| Lipf | 25.80 |  |  | |  |
| 9930020B22Rik | 27.81 |  |  | |  |
| LOC243428 | 28.95 |  |  | |  |
| Chi3l1 | 30.10 |  |  | |  |
| Krt1-14 | 51.43 |  |  | |  |
| E2f5 | 55.91 |  |  | |  |
| Saa3 | 57.34 |  |  | |  |
| 2300002M23Rik | 64.79 |  |  | |  |
| Lgr7 | 77.66 |  |  | |  |
| F10 | 78.18 |  |  | |  |
| Chi3l3 | 85.86 |  |  | |  |
| Ecel1 | 104.52 |  |  | |  |
| Retnlg | 104.74 |  |  | |  |
| Ugt1a1 | 150.87 |  |  | |  |
| LOC381568 | 169.38 |  |  | |  |
| Sprr2f | 169.92 |  |  | |  |
| C630016C03Rik | 174.43 |  |  | |  |
| E230022G02Rik | 204.10 |  |  | |  |
| Chad | 220.23 |  |  | |  |
| Pdrg1 | 307.24 |  |  | |  |
| Sspn | 1921.90 |  |  | |  |

S5 Table gives gene lists with significant differences of mRNA levels of the young and adult estrogen treated mouse models, relative to controls, obtained by cDNA analysis. Young mice subjected to cDNA microarray analysis, E2 Ratio indicated the estrogen treatment group signal value/control signal value; and in cDNA sequencing of the adult mouse model, E2 ratio indicated the logarithm of corresponding signal value ratios. About the bioinformatics analysis, described briefly as follows.

**cDNA chip for young mouse model:** ovarian tissues in Con group and E2 treated group, each from 10 mice, their respective RNA samples mixed into RNA pools were used for the DNA microarray analysis by United Gene Technology Co Ltd (Shanghai), Mouse WG-6 v2.0 chip (microarray chips, Illumina) was used as the mouse whole genome expression profiling bead chip. The three samples of this experiment were detected in at the same chip at one time, about 45200 transcripts were detected in each sample.

**cDNA sequencing for adult mouse model:** ovarian tissue in Con group and E2 group, each from 16 mice, mixed RNA samples were used for cDNA sequencing analysis. By Genergy Biotechnology Co Ltd, Shanghai, adopting single end sequencing of 50bp on Illumina Hiseq2500 sequencing platform.
